# The Implications of COVID-19 on Chinese Consumer Preferences for Lamb Meat

**DOI:** 10.3390/foods10061324

**Published:** 2021-06-08

**Authors:** Scott C. Hutchings, Luis Guerrero, Miranda Mirosa, Phil Bremer, Damien Mather, Enrique Pavan, Talia M. Hicks, Li Day, Carolina E. Realini

**Affiliations:** 1 AgResearch Limited, Te Ohu Rangahau Kai, Massey University Campus, Grasslands, Tennent Drive, Palmerston North 4474, New Zealand; Scott.Hutchings@agresearch.co.nz (S.C.H.); Enrique.Pavan@agresearch.co.nz (E.P.); Talia.Hicks@agresearch.co.nz (T.M.H.); Li.Day@agresearch.co.nz (L.D.); 2 IRTA-Monells, Finca Camps i Armet, 17121 Monells, Spain; Lluis.Guerrero@irta.cat; 3 Department of Food Science, University of Otago, P.O. Box 56, Dunedin 9054, New Zealand; miranda.mirosa@otago.ac.nz (M.M.); phil.bremer@otago.ac.nz (P.B.); 4 New Zealand Food Safety Science Research Centre, Hopkirk Research Institute, Tennent Drive, Massey University, Palmerston North 4442, New Zealand; damien.mather@otago.ac.nz; 5 Department of Marketing, University of Otago, P.O. Box 56, Dunedin 9054, New Zealand; 6 Instituto Nacional de Tecnología Agropecuaria, Balcarce 7620, Argentina

**Keywords:** COVID-19, lamb, meat, consumer, preference

## Abstract

This study assessed if Chinese consumer attitudes towards a range of lamb attributes (such as origin, food safety, appearance, taste, price), and their opinions of New Zealand lamb (9- and 7-point Likert scales, respectively), had changed since the outbreak COVID-19. The same survey was carried out in Shanghai and Beijing pre (December 2018) and post COVID-19 (November 2020), ~9 months after China’s initial outbreak, with 500 and 523 consumers, respectively. From December 2018 to November 2020, there was an increase in the proportion of Chinese consumers purchasing red meat online or from a butcher, and cooking their lamb well-done. In contrast, there were minimal differences in Chinese consumer ratings between December 2018 and November 2020 for different lamb attributes and opinions of New Zealand lamb. Cluster analysis revealed that many consumers (140 in December 2018/376 in November 2020) used only a small portion of the high end of the scale when rating lamb attributes, resulting in little differences between the attributes. This study suggests COVID-19 has enhanced some food safety related behaviors but had little effect on Chinese opinions and preferences for New Zealand lamb attributes. It also highlights that survey design should be carefully considered when collecting responses from Chinese consumers.

## 1. Introduction

As a result of the global COVID-19 pandemic, food producers and processors are faced with the possibility of changing consumer attitudes towards their products in markets all around the world. As well as COVID-19 being shown to commonly affect sensory acuity while people are infected with the disease [1], and in some cases after recovery [2], the pandemic has also been reported to change the way consumers view, interact, purchase, prepare and eat food [3,4,5]. In particular, recent studies have shown an increase in online purchasing of food [6,7,8,9], an increase in consumer demand for healthy and nutritious food [7,10], and an increase in consumer demand for long shelf-life food [3], since the COVID-19 outbreak. Consequently, there is a need for improved understanding of these changing attitudes and behaviours to assist producers and processers to remain competitive in the COVID-19 marketplace.

One food sector where it is particularly important to understand changes in consumer attitudes is the meat sector where food quality and safety are of paramount importance to consumers [11]. It has already been suggested that the COVID-19 pandemic has changed public awareness of illness linked to animals and altered meat consumption patterns, at least in the short term [12]. As a case study, the attitude of Chinese consumers to New Zealand lamb was selected for investigation. New Zealand lamb meat has historically had a reputation with international consumers as a safe, high-quality product, produced from a ‘clean and green’ environment [13]. Furthermore, lamb (and other red meats) have a longer shelf life than many other meats, such as poultry and fish [14]. Lamb is one of New Zealand’s most exported products, accounting for over NZD $3 billion in revenue each year [15], with China currently New Zealand’s largest importer of lamb meat [16]. China, the first country to suffer from the effects of COVID-19, and one of the world’s largest economies and largest importers of food, is a major market for most nations who export red meat [17].

One approach by which changes in Chinese consumer perception of New Zealand lamb meat can be measured before and after COVID-19 is through an online, quantitative consumer survey. In December 2018, approximately one year prior to the outbreak of COVID-19, AgResearch Ltd. completed an online quantitative survey with 250 Chinese consumers in Shanghai and 250 Chinese consumers in Beijing, on the importance of various meat attributes at the point of purchase and on the opinions of New Zealand lamb. Hence, for a timely assessment of consumer attitudes since the outbreak of COVID-19, an opportunity arose to repeat the same survey online with a similar number of consumers in the same location (Shanghai and Beijing) in November 2020.

The objectives of this study were, therefore, to determine if Chinese consumer attitudes towards a range of lamb attributes (such as animal origin, food safety, appearance, taste, price, brand), as well as their opinions of New Zealand lamb, have changed since the outbreak COVID-19, and if so how. It was hypothesized that since the outbreak of COVID-19, Chinese consumers would place more importance on the health, food safety, and price related attributes of lamb. It was also hypothesized that Chinese consumer opinions of New Zealand lamb as a nutritious/healthy, safe, and high-quality product would change.

## 2. Materials and Methods

### 2.1. Data Collection and Sample Characteristics

The survey in December 2018 involved recruiting five hundred consumers (*n* = 250 in Beijing and *n* = 250 in Shanghai), while the survey in November 2020 involved recruiting five hundred and twenty-three consumers (*n* = 265 in Beijing and *n* = 258 in Shanghai). Participants were recruited according to the following criteria: 18–75 years old, 50:50 male:female, and screening ensured that all recruited consumers ate lamb at least once per fortnight. A summary of the demographic characteristics of the four population groups is shown in Table 1.

The online survey in December 2018 was undertaken by the market research company COFCO Corporation (Beijing, China), while the online survey in November 2020 was undertaken by the market research company Dynata (Auckland, New Zealand). Both COFCO and Dynata used standard quality control techniques to ensure all responses were given by unique individuals without duplication or fraudulent responses. This survey was approved by the University of Otago Human Ethics Committee (Category B), application number D20/355.

### 2.2. Questionnaire

The questionnaire asked participants about a variety of demographic details, dietary and purchasing habits, lamb attributes of interest at the point of purchase and the type of lamb products they typically purchase. To gain insight into consumer considerations at the point of purchase, consumers rated the level of importance of varying aspects of lamb meat purchase on a scale of one (“not important”) to nine (“very important”). These aspects included animal factors and other production factors, pricing factors, intrinsic cues of the meat, convenience factors and personal knowledge of commercial cuts. To gain their opinion on New Zealand lamb meat, each consumer also rated their degree of agreement on several descriptions of the lamb meat on a scale of one (“strongly disagree”) to seven (“strongly agree”). New Zealand lamb meat was described in several ways, including, but not limited to, as nutritious, safe, good value for money, produced sustainably and convenient. The 9-point scale used to measure importance, and the 7-point scale used to measure opinions, are widely used forms of Likert scales for measuring consumer opinions of food [18,19].

The questionnaire was designed in English by the researchers before a native speaker of Mandarin translated the questionnaire into Mandarin. The Mandarin version included some small adaptations from the original English version to accommodate Chinese consumers. A complete copy of the English version of the survey can be found in Appendix A of this publication.

### 2.3. Data Analysis

IBM SPSS (V27) was used to analyze data. For data on demographic factors, diet and consumption patterns, a Chi-squared test was performed to firstly determine the effect of year (December 2018 vs. November 2020), and then the effect of city (Shanghai vs. Beijing). A two-way ANOVA was applied to the scale data on consumers’ rating of importance of lamb meat attributes at the point of purchase and their opinion on New Zealand lamb meat to determine the effect of year (December 2018 vs. November 2020), the effect of city (Shanghai vs. Beijing), and the effect of any year × city interaction.

An agglomerative hierarchical cluster analysis was performed on the square Euclidean distance matrix, with the Ward method, to identify three clusters of consumers based on their normalized scores for the level of importance of lamb attributes at the point of purchase using XLSTAT 2017 (Addinsoft 2012) software.

## 3. Results

### 3.1. Diet and Consumption Patterns of Consumers

Diet and consumption patterns were significantly different between the December 2018 and November 2020 consumers for all diets and meat types that were questioned (*p* < 0.05). A significant city effect was only found for dietary restrictions and for beef consumption (*p* < 0.05), showing higher proportions of consumers following low calorie diets in Beijing than Shanghai, and greater beef consumption in Beijing than Shanghai. Consumers in November 2020 were less likely to follow any particular diet. In general terms, November 2020 consumers ate beef and lamb slightly more often and pork, poultry and fish slightly less often than December 2018 consumers (Table 2).

### 3.2. Preferred Level of Cooking, Meat Qualities of Interest, Purchase Location and Types of Lamb Products Typically Purchased

Many of the preferences measured in terms of cooking, location of purchase, and types of lamb products typically purchased differed significantly between December 2018 and November 2020 consumers (*p* < 0.05) (Table 3). No city effect was found for any of the preferences measured (*p* > 0.05).

A greater proportion of the November 2020 consumers preferred their lamb well done compared to the December 2018 consumers. A much greater proportion of the November 2020 consumers purchased red meat online as well as at butcher shops compared to December 2018 consumers. Compared to December 2018 consumers, November 2020 consumers typically purchased less leg roast, chops, and shanks, and more lamb mince, rump and rack. All meat qualities of interest for December 2018 consumers were similar to November 2020 consumers (marbling, leanness, colour and portion size), with the exception of price which was looked for more by the December 2018 consumers (Table 3).

### 3.3. Importance of Lamb Attributes at the Point of Purchase (e.g., Origin, Food Safety, Appearance, Taste, Price)

There was no significant interaction ( *p* > 0.05) between year and city for consumers’ rating of the relative importance of lamb attributes at the point of purchase. The importance of most lamb attributes was significantly lower ( *p* < 0.05) with the November 2020 consumers compared to December 2018 consumers (both for Shanghai and Beijing) ( Table 4 ). However, the size of these effects was not large—the difference between year groups on average was only around 0.5 on the 9-point importance scale. The importance of lamb attributes did not differ significantly between the two cities studied ( *p* > 0.05).

Specifically, the November 2020 consumers showed significantly lower importance ratings ( *p* < 0.05) than December 2018 consumers for the following attributes: animal origin, animal welfare, animal feeding, animal age, presence of hormones and other residues, traceability, appearance, colour, flavour, texture, food safety, time of day to purchase, brand/quality label, labelling presentation, ease of preparation, dish to be prepared with, knowledge of different commercial cuts, and value for money. November 2020 consumers showed significantly higher importance ratings ( *p* < 0.05) for the sex of the animal, and the trust in the butcher. There was no significant difference between December 2018 and November 2020 consumers in importance ratings for lamb price, price of other meats, and place of purchase.

### 3.4. Opinion on New Zealand Lamb

Marginal year × city interactions were found for consumers’ opinion regarding lamb being healthy, well known, and convenient. Opinions towards New Zealand lamb were also significantly different between December 2018 and November 2020 consumers for a number of attributes, however, as observed with importance ratings for attributes of lamb, the size of any significant effects for year were relatively small—usually less than 0.5 on the 7 point Likert scale. Only two attributes showed marginally significant effects between cities (natural and no additives) (Table 5).

Specifically, November 2020 consumers’ opinion of New Zealand lamb was lower than December 2018 consumers for the following attributes: nutritious, healthy, safe, value for money, traditional product, natural, produced sustainably, high quality, contains no additives, makes people feel good, tastes good (*p* < 0.05). November 2020 consumers’ opinion of New Zealand lamb was higher than December 2018 consumers for hard to digest, boring, and readily available (*p* < 0.05). There was no difference between November 2020 and December 2018 consumers for well known, unique, and convenient (*p* > 0.05) (Table 5).

### 3.5. Cluster Analysis

**Figure 1:**
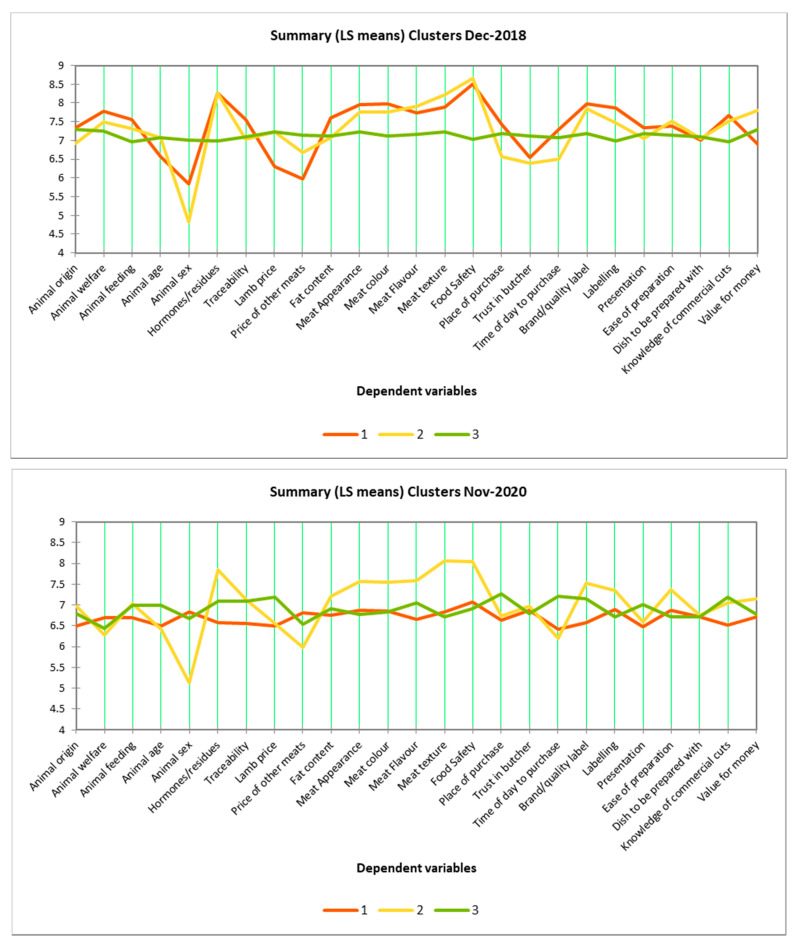
Cluster analysis of the importance ratings of lamb attributes at the point of purchase.

Cluster analysis of the December 2018 consumers (Figure 1) revealed three clusters: Cluster 1 (159 consumers), Cluster 2 (156 consumers), and Cluster 3 (185 consumers). In two of these clusters (Clusters 1 and 2) consumers assigned different importance ratings for the lamb attributes, while in Cluster 3 they gave virtually the same importance ratings for all attributes. December 2018 consumers in Clusters 1 and 2 assigned different importance ratings to attributes such as animal sex, hormones/residues, lamb price, price of other meats, meat appearance, colour, flavour, texture, food safety, trust in butcher, brand/quality label, labelling, knowledge of commercial cuts, value for money.

Cluster analysis of the November 2020 consumers (Figure 1) also revealed three clusters: Cluster 1 (224 consumers), Cluster 2 (152 consumers), and Cluster 3 (147 consumers). Here, consumers in one cluster (Cluster 2) assigned different importance ratings to the lamb attributes, while in the two other clusters (Clusters 1 and 3) they gave virtually the same importance ratings for all attributes. In November 2020, consumers in Cluster 2 assigned different importance ratings to lamb attributes such as animal sex, hormones/residues, lamb price, price of other meats, meat appearance, colour, flavour, texture, food safety, time of day to purchase, brand quality, labelling, and ease of preparation.

## 4. Discussion

### 4.1. The Effect of COVID-19 on Chinese Consumers’ Purchase Methods and Preferred Level of Cooking

This study has found that there was an increase in the proportion of consumers buying their red meat either online or from butcher shops (consumers that purchased meat online increased from 10.2% across the two cities in December 2018, to 29.1% in November 2020, and consumers that purchased meat from a butcher increased from 67% across the two cities in December 2018, to 78% in November 2020 (Table 3)). The increase in online purchasing reflects the global trend in increased online purchasing since COVID-19 [6,7], a trend that has also been shown in many Asian markets such as China [8] and Taiwan [9]. The increase in purchasing from butcher shops is also indicative of consumers trying to avoid going to supermarkets where there are large numbers of people, instead preferring a local butcher (interestingly, results also showed an increase in their trust of the butcher from December 2018 to November 2020 (Table 4)).

While Chinese consumers typically cook their meat in a style that is thorough and hence safe for consumption [20], this study has shown that from December 2018 to November 2020 the proportion of consumers wanting their meat well done increased and those wanting medium or medium/rare decreased (Table 3). This effect is likely a result of an implicit increase in consumer desire to prepare safe food for consumption following COVID-19. An online survey of 999 US consumers reported that the importance of food safety attributes of beef increased significantly following COVID-19 [21]. In terms of China, an online survey of 1008 consumers reported that COVID-19 increased their food safety concerns towards game meat [22]. In the current study, there was an increase in consumption of beef and lamb from December 2018 to November 2020, but no increase in the consumption of pork, poultry or fish (Table 2). The superior shelf life of red meat compared to poultry and fish [14], and the recent associations of pork with virus outbreaks [23,24] and unfounded fears of imported Salmon spreading COVID-19 [25], may have all contributed to beef and lamb becoming a more preferred option for home cooking by Chinese consumers in November 2020. Interestingly, fewer consumers were concerned with the price of red meat in November 2020, which may be linked to an implicit increase that Chinese consumers were placing on food safety over cost.

### 4.2. The Effect of COVID-19 on Chinese Consumer Responses towards the Importance of Lamb Attributes and Their Opinions of New Zealand Lamb

In general, consumer responses towards the importance of lamb attributes and opinions towards New Zealand lamb were largely unchanged from December 2018 to November 2020. Results, therefore, suggest that the COVID-19 pandemic has had only a very minor effect on Chinese consumer preferences towards lamb. For both the relative importance of lamb attributes, and the consumer opinions of New Zealand lamb, the slight reduction in ratings which were observed may have occurred as a result of other COVID-19 concerns (economic, health, family concerns) having greater priority for consumers. However, slight differences in the demographic characteristics or use of the scale between groups may be enough to explain these small effects.

Other literature that has recently been published in consumer science has shown an impact of COVID-19 on consumer attitudes towards food products in some cases. For example, consumers in Qatar have reported as having an increased desire towards healthier food products and local food products due to food safety concerns following the COVID-19 outbreak [7]. An online survey of 240 UK consumers looking at the effect of lockdown on their food attitudes, reported that after lockdown they placed more importance on health, mood, and weight control when choosing food, and less importance on familiarity [10]. In terms of purchase behaviour, an online survey of 362 Spanish consumers, reported that COVID-19 influenced their perceived purchase frequency of products across a wide range of product categories [3]. For example, they reported a decreased purchase frequency of short shelf life products such as fish and seafood, an increased purchase frequency of long shelf life products like pasta, and an increased purchase frequency of healthy products like vegetables and fruit. Perceived purchase frequency of meat also increased.

It is, however, important to note that the studies reported on above collected data from consumers at, or near, the height of the pandemic, and therefore, may not give an indication of consumer response to COVID-19 in the long term. When our study was conducted in November 2020, China had relatively low daily cases of COVID-19, city wide lockdowns had not been in place for many months, and the lifestyles of consumers had returned to a closer resemblance of normality in Shanghai and Beijing. The Chinese economy had also made a strong recovery by November 2020 [26]. Although the COVID-19 pandemic is still ongoing and many of the long-term consequences on consumers are still unknown, it is possible that with sufficient time many consumer habits with respect to food will return to normal [27].

### 4.3. Consumer Clusters, Limitations of This Study and Practical Implications of This Research

Cluster analysis revealed that 360/500 consumers (72%) in December 2018 and 152/523 consumers (29%) in November 2020 used a broad scale range and showed differential responses in importance ratings across numerous lamb attributes. However, the remaining clusters of consumers in December 2018 and November 2020 fell into a pattern of using a narrow scale range, effectively rating all lamb attributes at the point of purchase as important. While Asian consumers using a small scale range and higher on average scores in comparison to Western consumers is a common occurrence in sensory and consumer science [28,29,30], the large proportion of consumers who used such a narrow scale range was unexpected given that Likert scales have been used successfully in numerous studies in the past with Asian consumers [31,32]. The issue could be mitigated in future studies through the use of different types of survey methodologies, such as ranking based questions.

Results from this study, in particular comparisons with December 2018 and November 2020 consumers, are of course limited by the differences in demographic variables between the December 2018 and November 2020 consumers. Due to practical difficulties recruiting the same participants from the December 2018 survey, different consumers were recruited in November 2020. While recruitment ensured age and gender breakdowns were identical between years, other demographic characteristics (such as education, income, or other factors not measured) were not controlled for. A change in market research company used to recruit participants in December 2018 and November 2020 (which was also required for practical reasons), may have also contributed to small demographic differences between December 2018 and November 2020.

Finally, the results of this study provide assurance for producers and processors of lamb who export their products to China (especially those from New Zealand), that Chinese consumer preferences for lamb have not changed markedly since the outbreak of COVID-19. Exporters should feel confident that consumers in China who have valued the attributes of their lamb products in the past should continue to do so.

## 5. Conclusions

Between December 2018 and November 2020 (approximately nine months after the initial outbreak of COVID-19 in China) there was an increase in the proportion of Chinese consumers who buy their red meat online or at the butcher, and who cook their lamb to well-done, presumably as an implicit move towards safer food related behaviors. Interestingly, the importance consumers placed on a range of lamb attributes at the point of purchase and opinions towards New Zealand lamb did not vary over this time period.

Cluster analysis revealed that 140/500 consumers in December 2018 and 371/523 consumers in November 2020 used only a small scale range and thus assigned similar importance ratings to most lamb attributes raising some concerns about the suitability of the use of Likert scales for consumer research with Chinese consumers. Consequently, future research investigating the influence of COVID-19 or other food related topics on Chinese consumer attitudes should consider alternative survey methodologies to complement conventional scales to obtain greater discrimination across participants.

## Figures and Tables

**Table 1:** Demographic characteristics of consumers (%).

		Beijing December 2018	Shanghai December 2018	Beijing November 2020	Shanghai November 2020
Gender	Male	50.0	50.0	51.7	50.4
	Female	50.0	50.0	48.3	49.6
Age	18–25	13.2	12.8	12.8	12.8
	26–35	23.2	18.8	21.9	20.9
	36–45	13.6	18.4	15.5	16.3
	46–60	47.6	47.2	46.8	46.9
	61+	2.4	2.8	3.0	3.1
Education	none	0	0	0	0.4
	China elementary school	0	0	0	0.0
	Chinese junior high graduate	0.8	0.0	2.3	3.1
	Chinese high school	2.8	5.2	13.6	12.0
	Trades certificate	38.8	34.4	25.3	20.5
	Tertiary qualification	57.6	60.4	58.9	64.0
	Labourer	1.2	1.2	0.4	0.4
Income	Less than 50,000 CNY	1.2	1.6	0.4	0.8
	50,001 to 100,000 CNY	5.2	3.6	2.3	0.8
	100,001 to 150,000 CNY	17.2	13.2	10.6	7.0
	150,001 to 200,000 CNY	23.2	22.4	32.5	27.1
	200,001 to 300,000 CNY	32.0	39.2	24.2	33.7
	300,001 to 500,000 CNY	14.8	15.2	20.0	22.5
	More than 500,000 CNY	6.4	4.8	10.2	8.1

**Table 2:** Dietary restrictions and consumption frequency of animal protein sources (%) (*p* value determined using a Chi-squared test for both year and city).

		Beijing December 2018	Shanghai December 2018	Beijing November 2020	Shanghai November 2020	*p*(Chi^2^) (Year)	*p*(Chi^2^) (City)
Dietary restrictions	Low salt	72.8	73.6	63.8	60.9	<0.001	0.725
	Low sugar	71.2	72.8	65.7	59.3	0.001	0.413
	Low calories	63.2	61.2	52.8	40.7	<0.001	0.023
	Do not follow a diet	10.8	7.6	30.2	31.0	<0.001	0.571
Lamb	Daily	5.2	2.4	2.6	4.3	<0.001	0.110
	4–5 times a week	13.2	8.8	24.2	20.5		
	2–3 times a week	25.2	23.2	23.4	17.8		
	Weekly	35.6	40.4	32.1	37.6		
	Fortnightly	20.8	25.2	17.7	19.8		
Beef	Daily	3.2	2.0	10.6	4.7	<0.001	<0.001
	4–5 times a week	12.4	8.8	15.1	12.4		
	2–3 times a week	35.2	42.8	34.3	32.2		
	Weekly	32.4	38.0	24.9	29.8		
	Fortnightly or less	16.8	8.4	15.1	21.0		
Pork	Daily	14.8	12.4	11.7	11.2	<0.001	0.579
	4–5 times a week	26.0	26.4	25.3	19.0		
	2–3 times a week	39.6	40.0	31.3	34.1		
	Weekly	15.2	11.6	24.2	29.8		
	Fortnightly or less	4.4	9.6	7.6	5.8		
Poultry	Daily	8.0	4.4	6.0	2.7	<0.001	0.145
	4–5 times a week	13.2	12.0	14.7	11.6		
	2–3 times a week	37.2	40.8	39.2	35.7		
	Weekly	32.4	30.4	29.1	41.1		
	Fortnightly or less	9.2	12.4	10.9	8.9		
Fish	Daily	6.4	9.6	4.2	3.1	<0.001	0.774
	4–5 times a week	17.2	22.4	24.9	19.8		
	2–3 times a week	38.0	40.4	33.6	27.9		
	Weekly	32.4	18.0	27.9	38.0		
	Fortnightly or less	6.0	9.6	9.5	11.3		

**Table 3:** Preferred level of cooking, meat qualities of interest to consumers at the point of purchase and purchase and purchase frequency of different lamb products (%) (*p* value determined using a Fishers exact test (Chi-squared) for both year and city).

		Beijing December 2018	Shanghai December 2018	Beijing November 2020	Shanghai November 2020	*p*(Chi^2^) (Year)	*p*(Chi^2^) (City)
Preferred level of cooking with lamb	Rare	2.0	0.8	0.8	2.4	<0.001	0.133
	Medium/Rare	6.0	7.6	3.8	2.7		
	Medium	8.0	12.4	7.2	3.9		
	Medium/Well Done	53.2	58.8	35.1	39.1		
	Well Done	30.8	20.4	53.2	51.9		
Where do you purchase red meat?	Supermarket	92.4	91.6	84.5	90.7	0.023	0.150
	Butcher shop	66.4	66.8	80.0	75.6	<0.001	0.485
	Market	32.0	33.6	39.2	33.3	0.238	0.470
	On-line	8.0	12.4	26.4	31.8	<0.001	0.060
What qualities do you look for when purchasing red meat?	Marbling	52.4	50.4	52.5	55.0	0.491	0.950
	Leanness	87.6	85.2	85.7	86.0	0.857	0.718
	Meat colour	84.4	78.4	76.6	85.3	0.447	0.540
	Portion size	42.0	43.2	37.0	38.4	0.111	0.702
	Price	52.0	58.0	24.9	26.0	<0.001	0.539
What lamb products do you typically purchase?	Leg roast	68.4	64.0	56.6	60.5	0.012	0.967
	Lamb chops	56.8	59.2	40.8	46.9	<0.001	0.169
	Lamb mince	20.4	15.6	28.7	24.4	<0.001	0.085
	Lamb rump	32.0	29.6	38.5	41.1	0.003	0.975
	Lamb steaks	61.6	56.8	56.2	70.2	0.223	0.124
	Lamb rack	27.6	20.0	49.1	47.3	<0.001	0.119
	Lamb sausages	17.2	18.8	25.3	17.4	0.182	0.209
	Lamb shanks	51.2	57.6	44.2	45.3	0.002	0.235
	Shoulder roast	22.0	20.0	16.2	20.9	0.346	0.583

**Table 4:** The relative importance of lamb attributes at the point of purchase (mean ± SD) (1 = not important, 9 = very important). (*p* value determined using a two-way ANOVA with year and city as the main effects).

	Beijing December 2018	Shanghai December 2018	Beijing November 2020	Shanghai November 2020	*p* (ANOVA) (Year)	*p* (ANOVA)(City)	*p*(ANOVA) (City × Year)
Animal origin	7.12 ± 1.70	7.22 ± 1.47	6.80 ± 1.66	6.62 ± 1.64	<0.001	0.694	0.168
Animal welfare	7.52 ± 1.58	7.56 ± 1.35	6.44 ± 1.80	6.56 ± 1.69	<0.001	0.439	0.705
Animal feeding	7.23 ± 1.63	7.39 ± 1.49	6.88 ± 1.52	6.87 ± 1.48	<0.001	0.414	0.394
Animal age	6.84 ± 1.85	6.94 ± 1.62	6.75 ± 1.65	6.48 ± 1.64	0.010	0.465	0.080
Animal sex	5.70 ± 2.28	5.84 ± 2.21	6.34 ± 1.96	6.29 ± 1.78	<0.001	0.736	0.474
Presence of hormones and other residues	7.89 ± 1.47	7.98 ± 1.29	7.08 ± 1.71	7.09 ± 1.55	<0.001	0.635	0.652
Traceability (to know history of meat you purchase)	7.16 ± 1.74	7.33 ± 1.54	6.99 ± 1.63	6.76 ± 1.67	<0.001	0.782	0.052
Lamb price	6.85 ± 1.72	6.93 ± 1.70	6.73 ± 1.67	6.70 ± 1.59	0.094	0.813	0.597
Price of other meats	6.59 ± 1.83	6.48 ± 1.91	6.56 ± 1.77	6.44 ± 1.70	0.737	0.319	0.969
Fat content of meat	7.22 ± 1.52	7.35 ± 1.45	7.02 ± 1.48	6.83 ± 1.62	<0.001	0.747	0.095
General meat appearance (shiny, dry...etc.)	7.65 ± 1.36	7.75 ± 1.32	6.99 ± 1.57	7.09 ± 1.58	<0.001	0.274	0.998
Meat colour	7.60 ± 1.55	7.72 ± 1.29	7.06 ± 1.54	7.02 ± 1.63	<0.001	0.646	0.417
Meat flavour	7.57 ± 1.45	7.72 ± 1.21	7.06 ± 1.47	7.00 ± 1.53	<0.001	0.591	0.241
Meat texture (tenderness)	7.78 ± 1.47	7.91 ± 1.15	7.19 ± 1.53	7.09 ± 1.62	<0.001	0.875	0.197
Risk of catching a disease consuming lamb (food safety)	8.19 ± 1.37	8.17 ± 1.28	7.28 ± 1.53	7.31 ± 1.49	<0.001	0.952	0.776
Place of purchase	7.07 ± 1.56	7.01 ± 1.74	6.85 ± 1.63	6.86 ± 1.49	0.063	0.809	0.752
Trust in butcher	6.61 ± 1.69	6.66 ± 1.79	6.86 ± 1.62	6.90 ± 1.65	0.022	0.668	0.950
Time of the day in which you can purchase lamb	6.86 ± 1.94	7.03 ± 1.82	6.59 ± 1.71	6.58 ± 1.70	0.001	0.485	0.445
Brand or quality label	7.72 ± 1.37	7.72 ± 1.43	6.94 ± 1.65	7.08 ± 1.50	<0.001	0.474	0.423
Label information	7.42 ± 1.44	7.56 ± 1.47	6.96 ± 1.62	6.98 ± 1.53	<0.001	0.392	0.482
Presentation (pieces, slices, trays, etc.)	7.13 ± 1.58	7.25 ± 1.43	6.67 ± 1.70	6.67 ± 1.56	<0.001	0.533	0.551
Easy to prepare/cook	7.34 ± 1.43	7.39 ± 1.33	6.97 ± 1.60	6.97 ± 1.47	<0.001	0.814	0.806
Dish to be prepared with it	6.98 ± 1.72	7.10 ± 1.51	6.75 ± 1.56	6.71 ± 1.56	0.002	0.722	0.394
My knowledge of different commercial cuts	7.47 ± 1.46	7.40 ± 1.52	6.88 ± 1.55	6.84 ± 1.41	<0.001	0.581	0.823
Value for money	7.28 ± 1.57	7.40 ± 1.55	6.99 ± 1.61	6.72 ± 1.65	<0.001	0.448	0.050

**Table 5:** Opinion of New Zealand lamb (1 = strongly disagree, 7 = strongly agree) (mean ± SD) (*p* value determined using a two-way ANOVA with year and city as the main effects).

	Beijing December 2018	Shanghai December 2018	Beijing November 2020	Shanghai November 2020	*p* (ANOVA) (Year)	*p* (ANOVA)(City)	*p*(ANOVA) (City × Year)
Is nutritious	6.01 ± 0.99	6.13 ± 0.96	5.54 ± 1.03	5.58 ± 1.03	<0.001	0.208	0.474
Is healthy	5.92 ± 1.09	6.12 ± 0.95	5.68 ± 1.05	5.59 ± 1.00	<0.001	0.391	0.024
Is well known	5.52 ± 1.34	5.72 ± 1.25	5.59 ± 1.01	5.48 ± 1.07	0.214	0.549	0.034
Is unique	5.20 ± 1.40	5.43 ± 1.30	5.32 ± 1.22	5.38 ± 1.06	0.643	0.066	0.303
Is safe	5.89 ± 1.10	6.03 ± 0.99	5.57 ± 1.04	5.55 ± 1.06	<0.001	0.313	0.235
Is good value for money	5.59 ± 1.24	5.78 ± 1.02	5.51 ± 1.13	5.51 ± 1.06	0.015	0.182	0.175
Is boring	2.95 ± 1.83	3.11 ± 1.91	3.80 ± 1.95	3.59 ± 2.07	<0.001	0.834	0.136
Is a traditional product	5.22 ± 1.25	5.38 ± 1.18	5.18 ± 1.32	5.08 ± 1.36	0.036	0.707	0.105
Is natural	5.72 ± 1.23	5.98 ± 1.08	5.58 ± 1.00	5.60 ± 1.04	<0.001	0.047	0.070
Is hard to digest	3.40 ± 1.81	3.43 ± 1.94	4.25 ± 1.93	3.88 ± 1.99	<0.001	0.156	0.092
Is produced sustainably	5.66 ± 1.11	5.76 ± 1.12	5.48 ± 1.04	5.56 ± 1.04	0.005	0.184	0.830
Is convenient	5.33 ± 1.22	5.54 ± 1.15	5.49 ± 1.06	5.40 ± 1.08	0.857	0.408	0.034
Is readily available	5.08 ± 1.31	5.25 ± 1.14	5.36 ± 1.07	5.37 ± 1.20	0.007	0.210	0.285
Is high quality	6.02 ± 1.01	6.07 ± 1.01	5.65 ± 1.09	5.59 ± 1.05	<0.001	0.952	0.391
Contains no additives	5.58 ± 1.16	5.82 ± 1.06	5.53 ± 1.07	5.56 ± 0.99	0.022	0.044	0.131
Makes people feel good	5.92 ± 1.17	6.00 ± 0.96	5.53 ± 1.05	5.47 ± 1.11	<0.001	0.854	0.288
Tastes good	6.01 ± 0.93	6.05 ± 1.17	5.57 ± 1.07	5.67 ± 1.01	<0.001	0.269	0.664

## Data Availability

The data presented in this study are available on request from the corresponding author. Although consumer data have been anonymised, data are not publicly available.

## Appendix A

ID Number:

Consumer Lamb Study

Demographic Questionnaire

1. Gender

☐ Male	☐ Female

2. Age

☐ 18–25
☐ 26–35
☐ 36–45
☐ 46–60
☐ 61 and over

3. Please provide the postcode for where you currently live:

4. Please provide the location of where you grew up (e.g. rural location or name of suburb, town or city):
5. What is your highest level of education?

☐	None
☐	Primary school
☐	Middle School
☐	High School
☐	Trades certificate or vocational college
☐	Bachelor’s degree or higher

6. What is your occupation?

☐ Trades	☐ Home maker
☐ Professional	☐ Student
☐ Administration/Office	☐ Retired
☐ Sales/Services	☐ Unemployed
☐ Technical	☐ Other employment
☐ Labourer	

7. Which of these income levels best represents your combine household income (or personal if single) per annum?

☐ Less than 50, 000 CNY
☐ 50, 001 to 100, 000 CNY
☐ 100, 001 to 150, 000 CNY
☐ 150, 001 to 200, 000 CNY
☐ 200, 001 to 300, 000 CNY
☐ 300, 001 to 500, 000 CNY
☐ More than 500, 000 CNY

8. How many people live in your household (adults are aged 18 and over).

	1	2	3	4	5	6	7	8 (or more)
Adults	☐	☐	☐	☐	☐	☐	☐	☐
Children	☐	☐	☐	☐	☐	☐	☐	☐

9. Please indicate if you follow any of these diets (you may select more than one option).

☐ Low salt
☐ Low sugar
☐ Low calories
☐ Other—please specify______________
☐ I don’t follow any diets

10. How often do you consume the following types of meat?

Meat Type	Daily	4–5 times a week	2–3 times a week	Weekly	Fortnightly	Monthly	Never
Lamb	☐	☐	☐	☐	☐	☐	☐
Beef	☐	☐	☐	☐	☐	☐	☐
Pork	☐	☐	☐	☐	☐	☐	☐
Poultry	☐	☐	☐	☐	☐	☐	☐
Fish	☐	☐	☐	☐	☐	☐	☐

11. When consuming grilled lamb, what level of cooking do you prefer?

☐ Blue ☐ Rare ☐ Medium/Rare
☐ Medium ☐ Medium/Well Done ☐ Well Done

12. Where do you usually purchase red meat for your household?

☐ Supermarket
☐ Butcher shop ☐ Market
☐ On-line
☐ Other—please specify

13. What qualities do you look for when purchasing red meat? (Select all applicable)

☐ Marbling
☐ Leanness
☐ Meat colour
☐ Portion size
☐ Price
☐ Other- please specify _______________________

14. What lamb products do you typically purchase? (Select all applicable)

☐ Leg roast
☐ Lamb chops
☐ Lamb mince
☐ Lamb rump
☐ Lamb steaks
☐ Lamb rack
☐ Lamb sausages
☐ Lamb shanks
☐ Shoulder roast
☐ Other—please specify _______________________

15. Please circle a number that indicates the level of importance each aspect is to you when purchasing lamb.

1. Animal origin

1	2	3	4	5	6	7	8	9
Not Important								Very Important

2. Animal welfare

1	2	3	4	5	6	7	8	9
Not Important								Very Important

3. Animal feeding

1	2	3	4	5	6	7	8	9
Not Important								Very Important

4. Animal age

1	2	3	4	5	6	7	8	9
Not Important								Very Important

5. Animal sex

1	2	3	4	5	6	7	8	9
Not Important								Very Important

6. Presence of hormones and other residues

1	2	3	4	5	6	7	8	9
Not Important								Very Important

7. Traceability (to know history of meat you purchase)

1	2	3	4	5	6	7	8	9
Not Important								Very Important

8. Lamb price

1	2	3	4	5	6	7	8	9
Not Important								Very Important

9. Price of other meats

1	2	3	4	5	6	7	8	9
Not Important								Very Important

10. Fat content of meat

1	2	3	4	5	6	7	8	9
Not Important								Very Important

11. General meat appearance (shiny, dry...etc.)

1	2	3	4	5	6	7	8	9
Not Important								Very Important

12. Meat colour

1	2	3	4	5	6	7	8	9
Not Important								Very Important

13. Meat flavour

1	2	3	4	5	6	7	8	9
Not Important								Very Important

14. Meat texture (tenderness)

1	2	3	4	5	6	7	8	9
Not Important								Very Important

15. Risk of catching a disease consuming lamb (food safety)

1	2	3	4	5	6	7	8	9
Not Important								Very Important

16. Place of purchase

1	2	3	4	5	6	7	8	9
Not Important								Very Important

17. Trust in butcher

1	2	3	4	5	6	7	8	9
Not Important								Very Important

18. Time of the day in which you can purchase lamb

1	2	3	4	5	6	7	8	9
Not Important								Very Important

19. Brand or quality label

1	2	3	4	5	6	7	8	9
Not Important								Very Important

20. Label information

1	2	3	4	5	6	7	8	9
Not Important								Very Important

21. Presentation (pieces, slices, trays...etc)

1	2	3	4	5	6	7	8	9
Not Important								Very Important

22. Easy to prepare/cook

1	2	3	4	5	6	7	8	9
Not Important								Very Important

23. Dish to be prepared with it

1	2	3	4	5	6	7	8	9
Not Important								Very Important

24. My knowledge of different commercial cuts

1	2	3	4	5	6	7	8	9
Not Important								Very Important

25. Value for money

1	2	3	4	5	6	7	8	9
Not Important								Very Important

26. Others (indicate) __________________________

1	2	3	4	5	6	7	8	9
Not Important								Very Important

16. In your opinion New Zealand lamb……

	1	2	3	4	5	6	7
	Strongly disagree			Neither disagree nor agree			Strongly Agree
Is nutritious	☐	☐	☐	☐	☐	☐	☐
Is healthy	☐	☐	☐	☐	☐	☐	☐
Is well known	☐	☐	☐	☐	☐	☐	☐
Is unique	☐	☐	☐	☐	☐	☐	☐
Is safe	☐	☐	☐	☐	☐	☐	☐
Is good value for money	☐	☐	☐	☐	☐	☐	☐
Is boring	☐	☐	☐	☐	☐	☐	☐
Is a traditional product	☐	☐	☐	☐	☐	☐	☐
Is natural	☐	☐	☐	☐	☐	☐	☐
Is hard to digest	☐	☐	☐	☐	☐	☐	☐
Is produced sustainably	☐	☐	☐	☐	☐	☐	☐
Is convenient	☐	☐	☐	☐	☐	☐	☐
Is readily available	☐	☐	☐	☐	☐	☐	☐
Is high quality	☐	☐	☐	☐	☐	☐	☐
Contains no additive	☐	☐	☐	☐	☐	☐	☐
Makes people feel good	☐	☐	☐	☐	☐	☐	☐
Tastes good	☐	☐	☐	☐	☐	☐	☐
